# Pediatric Sarcoidosis: A Review with Emphasis on Early Onset and High-Risk Sarcoidosis and Diagnostic Challenges

**DOI:** 10.3390/diagnostics9040160

**Published:** 2019-10-25

**Authors:** Brian Chiu, Jackie Chan, Sumit Das, Zainab Alshamma, Consolato Sergi

**Affiliations:** 1 Department of Laboratory Medicine and Pathology, University of Alberta, Edmonton, Alberta T6G 2B7, Canada; jackiech@ualberta.ca (J.C.); sumit1@ualberta.ca (S.D.); zalshamm@ualberta.ca (Z.A.); sergi@ualberta.ca (C.S.); 2 Section of Neuropathology, University of Alberta, Edmonton, Alberta T6G 2B7, Canada; 3 Department of Paediatrics, University of Alberta, Edmonton, Alberta T6G 2B7, Canada

**Keywords:** Paediatric sarcoidosis, high-risk sarcoidosis, early-onset sarcoidosis, diagnostics, Blau syndrome

## Abstract

Sarcoidosis is a non-necrotizing granulomatous inflammatory syndrome with multisystemic manifestations. We performed a systematic review of sarcoidosis in the pediatric population with particular emphases on early onset sarcoidosis, high-risk sarcoidosis, and newly reported or unusual sarcoid-related diseases. Blau Syndrome and early onset sarcoidosis/ BS-EOS are seen in children younger than five years old presenting with extra-thoracic manifestations but usually without lymphadenopathy and/or pulmonary involvement. The prevalence of high-risk sarcoidosis is very low in children and is further limited by the difficulty of diagnosis in symptomatic children and underdiagnosis in subclinical or asymptomatic patients. Reports of sarcoidal syndromes in users of E-cigarette/marijuana/other flavorings and their induction in cancer immunotherapies are of interests and may be challenging to differentiate from metastatic malignancy. The diagnostic considerations in pediatric sarcoidosis are to support a compatible clinicoradiographic presentation and the pathologic findings of non-necrotizing granulomas by ruling out granulomas of infective etiology. There is no absolutely reliable diagnostic test for sarcoidosis at present. The use of endoscopic bronchial ultrasound (EBUS) and transbronchial fine needle aspiration (TBNA) sampling of intrathoracic lymph nodes and lung, and for superficially accessible lesions, with cytopathological assessment and pathological confirmations provide fair diagnostic yield and excellent patient safety profile in children.

## 1. Introduction

Sarcoidosis is a multisystemic syndrome with a highly variable clinical course and diverse disease manifestations [1]. The incidence of sarcoidosis in adults may be biphasic [2]. Historically, it was thought to commonly affect young adults 30–50 years of age, but recent studies have reported that more than half of incident diagnoses are made in patients over 55 years of age [2,3]. Erdal and others suggest that the rates of sarcoidosis are increasing [4]. Approximately 25% of affected individuals with the disease develop chronic and progressive disease, which contributes to increased disease burden [5,6]. The mortality rate also appears to be rising [7]. There is no single diagnostic test for sarcoidosis. Instead, the diagnosis relies on specific pathologic and radiographic features in the appropriate clinical settings. The disease is characterized by pathologic findings of non-necrotizing granulomas in one or more involved organ systems after alternative diagnoses, in particular, infective etiologies, have been entertained [8].

Sarcoidosis is an ever-evolving process. The clinical phenotypes range from single-organ, self-limited, asymptomatic disease to multi-organ involvement with high-risk manifestations [9]. Hilar lymphadenopathy and pulmonary interstitial infiltrations are the most common manifestations [10]. The term “high-risk sarcoidosis” was introduced at the National Heart Lung and Blood Institute Sarcoidosis Workshop 2017 [1] to denote several manifestations of sarcoidosis that are associated with impaired quality of life and relatively high risk of death [9]. These include treatment-resistant pulmonary sarcoidosis, cardiac sarcoidosis, neurosarcoidosis, and/or multi-organ involvement. The high-risk manifestations and multi-organ involvements are often missed until late in the disease course [9].

The diagnosis of sarcoidosis is relatively uncommon in children [8,11,12,13], and high-risk sarcoid may present differently in children than in adults [8,14]. In this review, we search the English literature and aim to review the clinical investigations and laboratory diagnostics of sarcoidosis in this population.

## 2. Materials and Methods

We performed a systematic narrative review on sarcoidosis with particular emphases on early onset sarcoidosis, high-risk sarcoidosis, and newly reported or unusual sarcoid-related diseases in the pediatric population. We searched PubMed, Scopus, Google Scholars, and Cochrane Database of Systematic Reviews, using the following terms: sarcoidosis, pediatric, juvenile, children; high-risk sarcoidosis; pulmonary sarcoidosis; treatment-resistant sarcoidosis; cardiac sarcoid; and neurosarcoidosis. We also searched references from the appropriate reviews and case reports.

## 3. Results

The true incidence and prevalence of sarcoidosis in children is unknown as the disease is much less common in children than in adults [13]. It is difficult to diagnose in symptomatic children and may remain undiagnosed in subclinical or asymptomatic patients [8,11]. Several larger reviews reported that the incidence of clinically recognized sarcoidosis in children was 0.22 to 0.29/100,000 children per year, and gradually increases with age to a small peak in teenagers at 13–15 years of age [2,8,11,13,15]. Two distinct forms of childhood sarcoidosis appear to exist. Older children and young adults present most frequently with a multisystemic disease in a combination of lymphadenopathy, pulmonary, ocular and cutaneous involvement (erythema nodosum) [2,8,11,13], followed by joint (sarcoidal arthritis) and hepatosplenic features [8,11,13]. The disease patterns and clinical outcomes are similar to those in adults.

### 3.1. Blau Syndrome and Early Onset Sarcoidosis (BS-EOS) 

In younger children, the majority of cases of EOS seen in children younger than five years old uniquely presents with a clinical triad of uveitis, arthritis, and skin rash [2]. In 1985, Blau et al. reported a rare autosomal dominant inflammatory disease characterized by a clinical triad of granulomatous dermatitis, recurrent granulomatous uveitis, and systemic polyarthritis in 11 family members of four generations [16,17]. A mutation of nucleotide binding oligomerization domain 2 (NOD2), also termed caspase recruitment domain–containing protein 15 (CARD15), mapped on chromosome 16q12 was identified [18] and found to be responsible for these granulomatous inflammations [16,18]. Early onset sarcoidosis (EOS) is caused by a sporadic mutation of the NOD2 gene, with similar clinical manifestations to Blau Syndrome [16]. Blau Syndrome/EOS patients rarely present with pulmonary involvement, and ocular involvement tends to follow prior articular and cutaneous manifestations in these patients [16,19].

CARD15/NOD2 encodes a multidomain cytosolic protein and is expressed primarily in the cytosol of antigen-presenting cells [20]. Stimulation by bacterial or viral infection [21] may promote the activation of nuclear factor kappa-light-chain-enhancer of activated B cells (NF-κB) and tumor necrosis factor receptor-associated factor 3 (TRAF3) signaling pathways [21]. A possible role of mycobacterial components has also been suggested as triggers of granulomatous autoinflammation in CARD15/NOD2 mutations in BS/EOS [22,23,24]. Crohn’s disease (CD) is a granulomatous inflammatory bowel disease, involving any part of the gastrointestinal tract [25] but may mainly affect the distal ileum and colon [22,26,27]. The CARD15/NOD2 gene has been identified as one of the genes linked to susceptibility to CD [22,28]. It has been hypothesized that NOD2 susceptibility loci in intestinal Paneth cells led to defective NF-κB activation, altered intestinal bacterial clearance and granulomatous inflammation [22,29,30]. Juvenile sarcoidosis presenting as Crohn’s disease of the GIT has been reported [31].

### 3.2. High-Risk Sarcoidosis 

Approximately 25% of affected individuals with sarcoidosis develop chronic or progressive disease [6]. Manifestations of sarcoidosis associated with poor prognosis and a relatively high risk of death include treatment-resistant pulmonary-, cardiac-, neuro-, and multiorgan sarcoidosis [9]. Pulmonary disease is the most common cause of chronic disease and death in adults [9]. As in adults, older children usually present with hilar lymphadenopathy and up to 50% present with interstitial lung disease and multiorgan involvement, while progression to chronic diseases occurs in 12% of these children [2,8,13]. Korsten et al. defined pulmonary sarcoidosis refractory to treatment as progressive disease and significant impairment of life despite glucocorticoid therapy for at least three months and the need for additional anti-sarcoid drugs [32]. These patients develop progressive pulmonary fibrosis and associated complications, including pulmonary hypertension and infections and are seen in more than 10% of patients at specialized centers [32,33]. In infants and children younger than five years, the typical pulmonary diseases are usually not seen [8]. In a retrospective study of 41 pediatric patients, which were followed for 18 months on disease presentation, management, and clinical outcome, those patients with pulmonary sarcoidosis diagnosed before 10 years old were more likely to recover and presented with fewer relapses compared with the patients diagnosed after 10 years old [34].

Recent progress in cell-mediated immunity and granuloma formation has advanced our understanding of the development of sarcoidosis [8,35]. Macrophages bearing increased expression of major histocompatibility (MHC) class II molecules, different subsets of T-lymphocytes and other immune effector cells such as mast cells and natural killer cells may be at play [8,35]. In sarcoidosis involving the lung, before the formation of granulomas, early lesions consist of alveolitis with a high proportion of activated CD4+ T-cells [36]. T-cells play a role in amplification of the local cellular immune response and are responsible for secretion of cytokines, including tumor necrosis factor (TNF), which favors granulomatous response at the sites of disease activity. The diagnostic approach to pulmonary sarcoidosis has been revolutionized in the past decade by the use of endobronchial ultrasound (EBUS) real-time guided transbronchial sampling of intrathoracic lymph nodes and lung biopsies [6]. Cytopathological assessments by fine needle aspiration provide fair diagnostic yield and excellent patient safety profile in children [37,38]. Combining EBUS and transbronchial needle aspiration (TBNA) lung biopsy and cytopathologic study may increase the diagnostic sensitivity to close to 100% [39,40]. On bronchoalveolar lavage (BAL) fluid with lymphocytosis (15%) and increased ratio of CD4 to CD8 (>3.5:1), the specificity for the diagnosis of sarcoidosis approaches 95% [36,41]. 

Cardiac sarcoidosis (CS) is a rare but potentially fatal condition [42]. Clinically recognizable cardiac involvement occurs in 5% of adult patients with sarcoidosis [43], and granulomatous inflammation of the heart was recognized in up to 25% in an autopsy series [13,44]. Rare cases of pediatric cardiac sarcoidosis had been reported [8]. Patients with CS may present with a wide variety of signs and symptoms, ranging from asymptomatic ECG abnormalities to sudden death [42]. Congestive heart failure and conduction abnormalities are the two most common clinical manifestations in CS [42], with one case presenting with pericardial effusion [2,8]. Many patients with pulmonary/systemic sarcoidosis have asymptomatic cardiac involvement [43]. A high index of suspicion and early diagnosis are crucial when immunosuppression therapy may result in reduced mortality rate [8]. Various imaging modalities including echocardiography and/or CMR imaging may be utilized [43].

Neurosarcoidosis occurs in fewer than 5% of adult with systemic sarcoid with isolated neurosarcoidosis being more rare [45], and to our knowledge, 53 cases of pediatric neurosarcoidosis have been reported in the English literature [14,46]. In these children, the most common manifestations include headache, seizures, cranial neuropathy, optic neuritis, and hypothalamic and/or pituitary dysfunction [14,46]. Compared to the adult counterparts, prepubertal children with neurosarcoidosis are more likely to present with seizures and a space-occupying lesion but less likely with cranial nerve palsies. These children do however tend to evolve to an adult pattern of presentation during puberty [14]. Single cases of aqueductal stenosis [13] and obstructive hydrocephalus [47] have also been reported. The most reliable method for diagnosing neurosarcoidosis is via biopsy of lesion in the central nervous system revealing non-caseating granuloma [46]. Microscopically, granulomatous inflammation is typically found in the meninges, ventricles (including choroid plexus) and adjacent brain or spinal cord parenchyma. Inflammatory infiltrate may involve optic nerve and chiasm, cranial nerves such as facial, auditory, and vestibulocochlear nerve. In cases where biopsy is not possible, neuroimaging becomes an important diagnostic modality. The most common neuroimaging finding is said to be leptomeningeal enhancement on T1-weighted MRI with contrast administration [48]. Enhancement may also be seen in the basilar region, leading to abnormalities in the hypothalamus, optic chiasm, and pituitary region [46]. As neurosarcoidosis is a diagnosis of exclusion, differential diagnoses that need to be considered and ruled out include tuberculosis, fungus, Wegener’s granulomatosis and hypertrophic meningitis, as these may also present with leptomeningeal enhancement and granulomas. To the best of our knowledge, cases of pediatric spinal neurosarcoidosis have not been reported in the English literature.

### 3.3. Acute Sarcoidosis 

In contrast to the chronic progressive disease with organ dysfunction in high-risk sarcoidosis, two acute sarcoidosis syndromes are of relatively benign clinical outcome. Lofgren syndrome is characterized by fever, bilateral hilar lymphadenopathy, erythema nodosum, and arthralgia, typically in young women, primarily Caucasian. Prognosis is excellent with complete resolution of the disease in 90% of the patients within two years [8,49,50]. Heerfordt syndrome or uveoparotid fever, presenting with a combination of fever, parotidomegaly, anterior uveitis, and cranial nerve palsy is usually seen in adults but has been reported in children [8,49]. The disease is normally self-limiting with cure achieved between 1–3 years [8,51]. The acute sarcoidosis syndromes may be confidently diagnosed on clinical grounds without surgical biopsy confirmation in adults [8,49,52]. However, the clinical manifestations in children may be variable necessitating biopsy histopathology confirmation [8,15,49,53]. 

**Figure 1:**
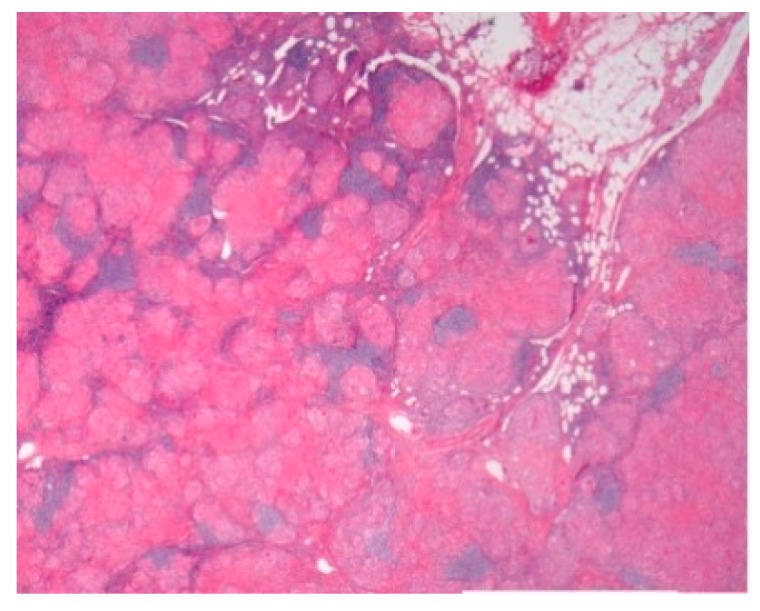
Mediastinal nodal sarcoidosis. H&E Magnification 20×.

**Figure 2:**
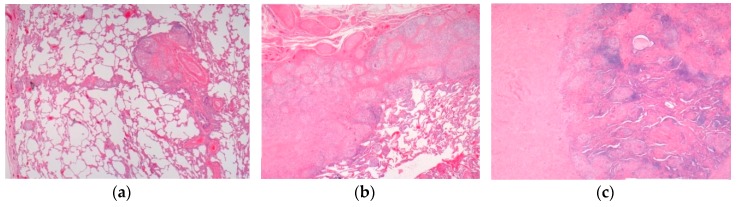
Pulmonary sarcoidosis. (**a**) Pulmonary sarcoidosis. (**b**) Pulmonary nodular sarcoidosis. (**c**) Pulmonary fibrotic sarcoidosis. H&E. Magnifications 20×, 50×.

**Figure 3:**
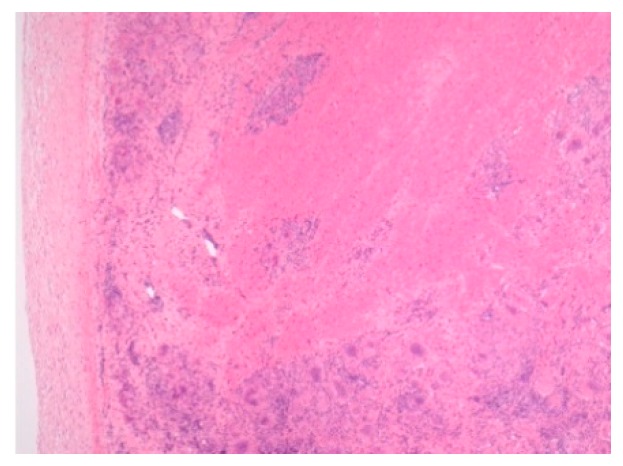
Cardiac sarcoidosis. H&E Magnification 50×.

**Figure 4:**
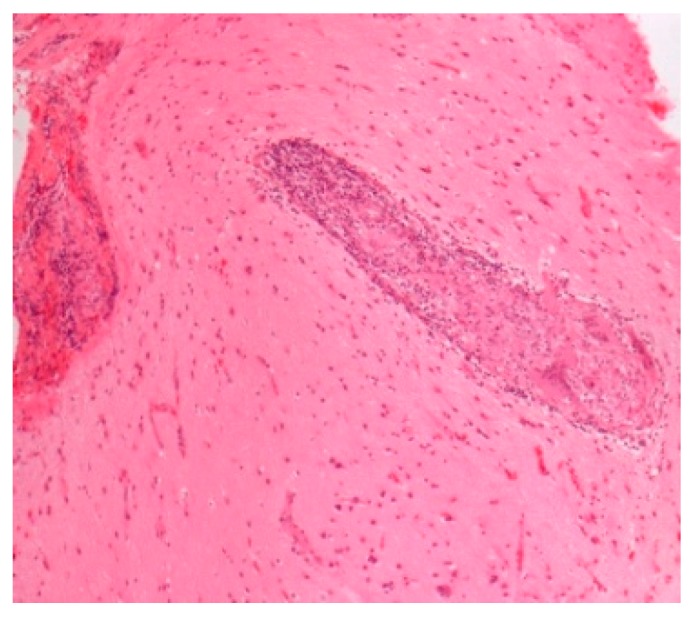
Neurosarcoidosis. H&E. Magnification 100×.

**Figure 5:**
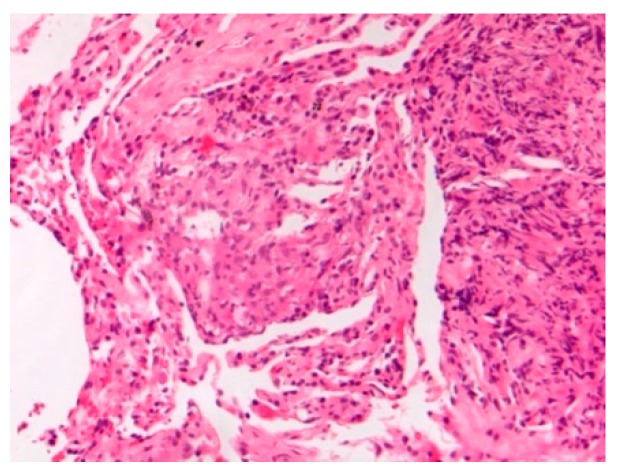
Lung sarcoidosis young E-cig vaper. H&E. Magnification 200×.

**Figure 6:**
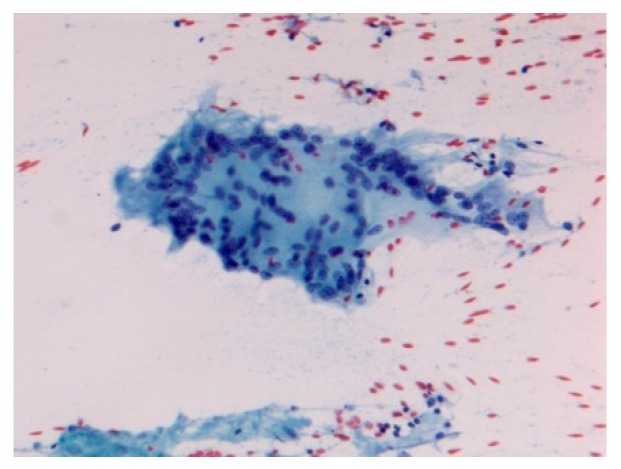
Acute sarcoidosis salivary gland. Cytopathology Pap stain 200×.

**Figure 7:**
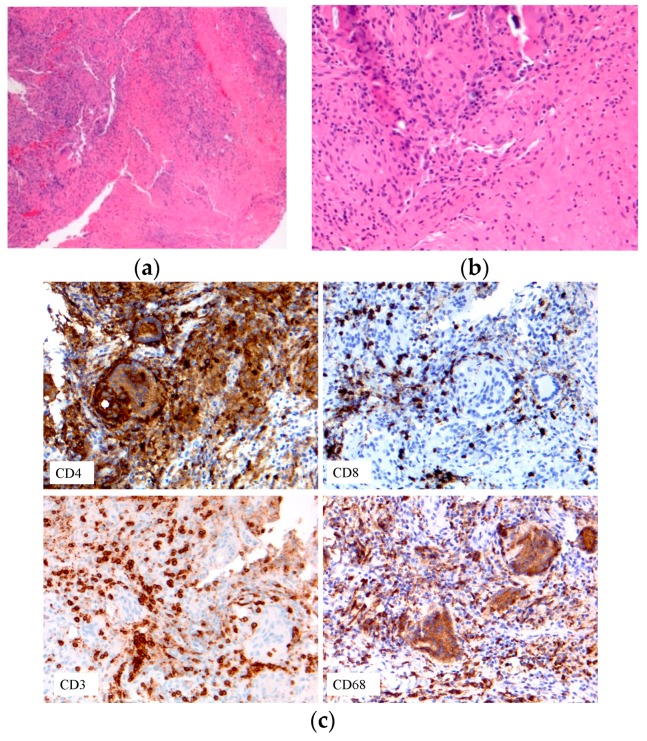
Immune cell phenotypes: T-cells: CD3, CD4, CD8; macrophage CD68. (**a**) Early onset sarcoidosis (EOS) skin sarcoidosis; (**b**–**c**) EOS skin sarcoidosis.

Pathology images of the spectrum of pediatric sarcoidosis involving different organs (Figure 1, Figure 2, Figure 3, Figure 4, Figure 5, Figure 6 and Figure 7). 

Recent case reports of sarcoidosis developing after antigen exposure to inhalation or in immune-therapy are of interests. The popularity of electronic cigarettes (E-cig) has recently been increasing among adolescents and young adults [54,55,56]. E-cig usage or “vaping” contains E-liquid carrier components, flavorings, and marijuana product with cannabidiol (CBD) formulations [57,58,59,60]. In anecdotal stories, the use of medical marijuana, in particular vaping with CBD oil may be linked to improvement of refractory pulmonary sarcoidosis [61]. In E-cig and marijuana users, sarcoidal granulomas are found in multiple organs in case reports, raising the clinical suspicion of metastatic malignancy [62,63]. The development of sarcoidal granulomas may be related to vaped marijuana product tetrahydrocannabinol (THC), E-cig flavorings, or contaminants. An association of vaped THC has been reportedly seen in close to 500 cases of severe pulmonary disease with six confirmed deaths [56]. As tumor necrosis factor-alpha (TNF-α) plays an important role in both formation and maintenance of sarcoidal granulomas, anti-TNF-α may provide a therapeutic option to patients with sarcoidosis [64]. However, many anti-TNF-associated cases of sarcoidosis have also been reported [65], negating its therapeutic usage. Pulmonary sarcoidal granulomas are found in patients undergoing interferon-alpha therapy [66]. The use of immune checkpoint inhibitors by enhancing anti-tumor immunity has revolutionized cancer therapy recently, and their use for cancer immunotherapy in pediatric patients may have potential benefits [67,68]. However, pulmonary sarcoidosis and the exacerbation of sarcoidosis leading to central nervous system involvement after immune checkpoint inhibitor therapy have been reported [25,69].

### 3.4. Diagnostic Challenges and Considerations in Pediatric Sarcoidosis 

There is no absolutely reliable diagnostic test for sarcoidosis [52]. A number of clinical tests and biomarkers assay have evolved over time in the diagnosis and include: tubulin skin test of anergy (Kvim test), serum angiotensin-converting enzyme (SACE), serum amyloid A (SAA), and cytokine levels [33,52,70]. The lack of sensitivity and specificity of these tests or biomarkers poorly suited as screening tests, disease activity or prognostic markers for sarcoidosis [33,52,70]. As in adults, the diagnosis is difficult to confirm in symptomatic children, and there is a significant number of asymptomatic patients who remain undiagnosed [13]. 

In the spectrum of pediatric sarcoidosis, depending on the initial clinical presentation and subsequent investigations including various diagnostic imaging modalities, sarcoidosis is a diagnosis of exclusion, with ruling out granulomatous infections, in particular by Mycobacterial and fungal organisms [8,52]. A high index of suspicion is critical in the diagnosis, especially in early onset of the disease or Blau syndrome in a child younger than five years of age who presents with a clinical triad of uveitis, arthritis, and skin rash, and without the adult manifestations of lymphadenopathy and/or pulmonary involvement. The early presentation of arthritis in early onset sarcoidosis may be misdiagnosed as juvenile rheumatoid arthritis (JRA) [71]. Pediatric sarcoidosis involving the gastrointestinal tract may raise a differential diagnosis mostly with Crohn’s disease [31]. Both sarcoidosis and CD are chronic granulomatous inflammatory disorders. The presence of granulomas in the gastrointestinal tract might be misleading and the diagnosis of sarcoidosis should be established when extraintestinal features become evident [31]. For BS/EOS and acute sarcoidosis syndromes, pathological and/or cytopathological confirmation and exclusion of other etiologies by biopsies of accessible lesions such as skin (including erythema nodosum, which is a non-granulomatous panniculitis) [49], salivary glands, and peripheral lymph nodes are highly desirable. 

**Figure 8:**
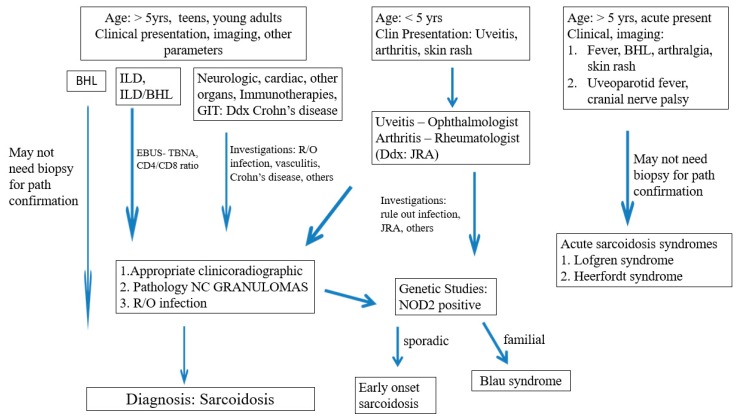
Paediatric Sarcoidosis: Diagnostic Considerations. Legends: BHL Bilateral hilar lymphadenopathy, ILD Interstitial lung disease, GIT gastrointestinal tract, EBUS-TBNA endoscopic bronchial ultrasound-transbronchial needle aspiration, NG non-necrotizing granulomas, JRA Juvenile rheumatoid arthritis, NOD2 Nucleotide binding oligomerization domain 2.

Cases of high-risk sarcoidosis in children with the involvement of treatment-resistant pulmonary disease, cardiac sarcoidosis, neurosarcoidosis, and multi-organ disease and the recent reports of E-cigarettes and immunotherapy-related sarcoidosis are uncommon. The lung is the most frequent site for biopsy confirmation, followed by skin, peripheral lymph nodes, and liver [49,72]. The diagnostic considerations are to support a compatible clinicoradiographic presentation and the demonstration of non-caseating granulomas. The use of EBUS-TBNA sampling of intrathoracic lymph nodes and lung [6], with cytopathological assessments and pathological confirmations provide fair diagnostic yield and excellent patient safety profile in children [6,52] (Figure 8).

## 4. Discussions

**Figure 9:**
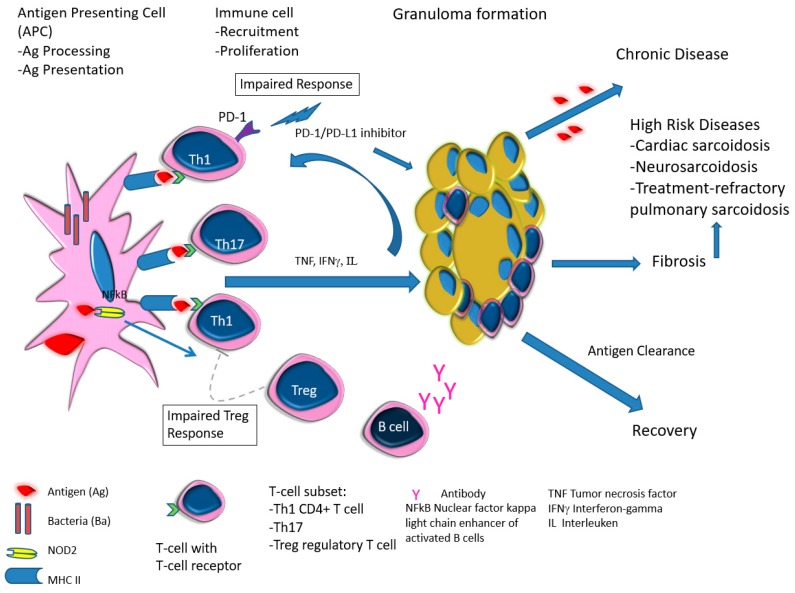
Proposed Pathogenetic Mechanisms in Pediatric Sarcoidosis.

Since the initial clinical description by Jonathan Hutchinson [73] in 1877, followed by the histologic findings of Caesar Boeck [74] in 1899, recent progress in immunology and molecular biology has advanced our understanding of the pathogenetic mechanisms for the development of sarcoidosis [8,35,75]. The proposed model of sarcoidal granuloma development in children involves the interplay of environmental triggers by a variety of antigens and immunologic/genetic/epigenetic factors, and requires three or more events to take place [8] (Figure 9)—environmental exposures to antigen (e.g., infectious agents, E-cigarettes flavorings, marijuana), acquired cellular immunity directed against the antigen, and the appearance of immune effector cells that promote a non-specific inflammatory response [8]. The subsequent Th1 (CD4+ T cell) polarized immune response with increased cytokine activities (TNF, IFNγ, interleukin) [75], and high-level IFNγ-secreting Th17 effector cells [35] are at play in the recruitment and proliferation of immune cells. Genetic factors (e.g., CARD15/ NOD2 mutation) may play a role by interacting with mycobacterial/bacterial/viral components, promoting the activation of NF-κB and tumor necrosis factor receptor-associated factor 3 (TRAF3) signaling pathways and triggering granulomatous autoinflammation in BS/EOS patients [22,23,24]. Immune checkpoint inhibitor (PD-1/PD-L1) for the immunotherapy of a variety of cancers may activate T-cells and promote granuloma formation [25,69]. Progression of granulomatous inflammation and putative antigen persistence promotes fibrosis and the development of high-risk organ-system manifestations [35,75].

Lofgren syndrome patients develop acute sarcoidosis, and show reduced frequency of lung-resident regulatory T cells (T-reg). The T-reg, however, super-synthesizes interleukin IL10, as supported by the detection of increased BAL fluid IL10 concentration [76], thus, creating an immunosuppressive micro-environment [35]. B lymphocytes may also be involved in sarcoidal granuloma formation, as Lofgren syndrome patients exhibit higher levels of Proprionibacterium acnes-specific IgA antibodies [77]. In chronic diseases, sarcoid T-reg fails to efficiently suppress proliferation and cytokine production by T helper cells [78], and with accumulation of Th1/Th17 cells, further support stabilization of granulomas and chronic disease progression [35]. In contrast, highly plastic and qualified effector T-cells aiming at clearance of the antigens from the granulomas leads to recovery [35,76]. 

We summarize the clinicopathologic spectrum of pediatric sarcoidosis and the diagnostic considerations in Table 1. Erdal and others suggest that the rates of adult sarcoidosis are increasing [4]. With the risks of increasing environmental exposure to increasing number of antigens, we would expect the prevalence of pediatric sarcoidosis also correspondingly increases. At present, there are no absolute diagnostic tests or biomarkers for screening, disease activity or prognostic markers for sarcoidosis. Fortunately, the diagnosis of sarcoidosis in children is improving with ever improving diagnostic techniques and imaging modalities. The development of minimally invasive procedures including EBUS-TBNA, for cytopathology or pathological confirmation of non-necrotizing granulomas and the resultant improving prognosis outcome appear promising. Recent advances in the use of genome-based approaches in identifying novel biomarkers to support the diagnosis and predict disease activity yield some promising results [70]. However, further researches are needed in sarcoidosis involving the young population. 

## Figures and Tables

**Table 1:** Pediatric sarcoidosis—clinicopathologic spectrum.

Entity	Clinical	Laboratory	Pathology	References
-Prevalence -Systems involved -Prognosis	-0.22–0.29/10^6^ -Lymph nodes/Lung -Chronic disease 12%	BAL: CD4/CD8 Biopsies	Granulomas Figure 1	[2,8,11,13,15]
-Early-onset sarcoidosis/ Blau syndrome	Triad: uveitis, arthritis, skin rash	Eye exam Biopsy	Granulomas Figure 7	[22,23,79]
High-risk sarcoid -Treatment-resistant Pulmonary sarcoid -Cardiac sarcoid/CS -Neurosarcoid/ NS	-Most common, progression to chronicity -CS Case reports -NS 53 cases	Chest XR, CT, EBUS/TBNA Cardiac echo CT /MRI	Figure 2 Figure 3 Figure 4	[2,9,13]; [2,6,8,14,34,46]
Sarcoid-like syndrome -INF therapy -Checkpoint inhibitor -E-cig/Marijuana	-Not reported in children -Not reported in children -Teens and young adults	Imaging modalities Biopsy	Figure 5	[66] [25,69]; [62,63]
Acute sarcoidosis -Lofgren syndrome -Heerfordt syndrome /uveoparotid fever	Triad: erythema nodosum BHL (CXR), arthritis, Resolution in two years Uveitis, parotidomegaly, facial nerve palsy, fever, Prognosis excellent	Chest XR, CT, biopsy	Both can be Diagnosed on clinical data without biopsy Figure 6	[50]; [8,49]

BAL bronchoalveolar lavage, EBUS endoscopic transbronchial ultrasound biopsy, TBNA transbronchial needle aspiration, IFNα interferon-alpha therapy, CPI checkpoint inhibitor therapy, Electronic-cigarette E-cig, BHL (CXR) bilateral hilar lymphadenopathy on chest X-ray.
